# Comparing herbaceous plant communities in active and passive riparian restoration

**DOI:** 10.1371/journal.pone.0176338

**Published:** 2017-04-27

**Authors:** Elise S. Gornish, Michael S. Lennox, David Lewis, Kenneth W. Tate, Randall D. Jackson

**Affiliations:** 1 Department of Plant Sciences, University of California Davis, Davis, CA, United States of America; 2 University of California Cooperative Extension, Marin/Sonoma/Mendocino Counties, Novato, CA, United States of America; 3 Department of Agronomy, University of Wisconsin-Madison, Madison, WI, United States of America; USDA-ARS Fort Keogh Livestock and Range Research Laboratory, UNITED STATES

## Abstract

Understanding the efficacy of passive (reduction or cessation of environmental stress) and active (typically involving planting or seeding) restoration strategies is important for the design of successful revegetation of degraded riparian habitat, but studies explicitly comparing restoration outcomes are uncommon. We sampled the understory herbaceous plant community of 103 riparian sites varying in age since restoration (0 to 39 years) and revegetation technique (active, passive, or none) to compare the utility of different approaches on restoration success across sites. We found that landform type, percent shade, and summer flow helped explain differences in the understory functional community across all sites. In passively restored sites, grass and forb cover and richness were inversely related to site age, but in actively restored sites forb cover and richness were inversely related to site age. Native cover and richness were lower with passive restoration compared to active restoration. Invasive species cover and richness were not significantly different across sites. Although some of our results suggest that active restoration would best enhance native species in degraded riparian areas, this work also highlights some of the context-dependency that has been found to mediate restoration outcomes. For example, since the effects of passive restoration can be quite rapid, this approach might be more useful than active restoration in situations where rapid dominance of pioneer species is required to arrest major soil loss through erosion. As a result, we caution against labeling one restoration technique as better than another. Managers should identify ideal restoration outcomes in the context of historic and current site characteristics (as well as a range of acceptable alternative states) and choose restoration approaches that best facilitate the achievement of revegetation goals.

## Introduction

Rapid changes in climate and land use are contributing to deteriorating structure and function of ecosystems [1–2]. Active restoration of plant communities is a growing component of preparing for, and limiting the loss of functional groups that are key to providing important ecosystem services [3–4]. However, despite enormous efforts, current active restoration approaches—including re-vegetation, soil remediation and invasive species management—have demonstrated limited success for stemming plant species extinctions and replenishing plant species losses [5]. This widely acknowledged inadequacy of many restoration strategies is largely driven by prohibitively high costs, and to a lesser degree, infeasible logistics [6]. For example, cost-effective restoration strategies that require considerable investment in terms of equipment or manpower will likely not be adopted for widespread use.

Passive restoration reduces or eliminates anthropogenic environmental stressors [7] such as grazing, development, or logging, and has shown promise in degraded forests [8–9], streams [7], sagebrush steppe [10], and riparian habitat [11]. This approach is typically less costly and more manageable than active restoration, but might not be as effective [11–12]. Understanding the relative efficacy of passive and active restoration should improve the success of restoration strategies, but studies explicitly comparing active and passive restoration outcomes are rare [13].

Riparian habitats are excellent systems for exploring restoration success because both active and passive restoration approaches have demonstrated utility in these environments [14–16]. For example, maintenance and enhancement of bank stability through active restoration, such as tree plantings and subsequent herbaceous plant community reestablishment, is one of the dominant goals of riparian restoration [17] because bank stability affects almost all other ecosystem services associated with riparian systems. These services include instream heterogeneous habitat development, water clarity and nutrient loads, and channel formation [18]. However, since the presence and activities of grazing animals (both livestock and wildlife) can significantly exacerbate erosion through soil compaction and vegetation loss, restoration through the temporary and periodic exclusion of grazing animals is a common management technique for riparian restoration [19–20] but see [21–22]. In the absence of restoration activities such as planting, grazing exclusion in degraded riparian areas is considered passive restoration. However, passive restoration usually incurs significant costs for fencing, forage loss, and water management [23,12]. Moreover, grazing removal from riparian areas may increase nutrient loss from terrestrial to aquatic ecosystems [24] and result in undesirable plant community change [22–23].

Assessing restoration outcomes through basic observational studies of long-term projects is key to evaluating the efficiency of alternative approaches to riparian restoration, e.g. [13–14, 25]. We investigated the efficacy of active and passive riparian corridor restoration on California’s north coast. We measured biophysical attributes of riparian sites varying in age since restoration and revegetation technique (i.e., active or passive). These sites were located along tributary stream reaches in California across a range of years since revegetation. Previously we analyzed results from these sites for trajectories in plant community and aquatic habitat metrics [15]. Here, we asked (1) How does restoration success differ between actively and passively restored sites? and (2) How does restoration type interact with site characteristics to affect herbaceous plant communities? Despite the well-documented relationship between riparian vegetation composition and underlying abiotic factors [26–27], we expected that herbaceous communities would depend on restoration type [28] considering how profoundly these communities can change following management of riparian systems [29].

## Materials and methods

### Study sites

We surveyed 103 riparian sites once each during the summers between 2003 and 2005 in a three-county (Marin, Sonoma, and Mendocino) study area in California U.S., dominated by oak woodlands and annual grasslands [15]. The region is characterized by a Mediterranean climate with cool, wet winters and hot, dry summers. During the study period, mean annual precipitation in the region was 101 cm and mean annual temperature was 13.7°C. Streams and rivers in the study area are dominated by varying degrees of channel incision and are located in watersheds with an average area of 23.5 km^2^. Surveyed sites were primarily on first-, second-, and third-order streams that were exposed to restoration between 0 and 39 years ago. Ninety of the 103 surveyed sites were restored. At passively restored sites (39 out of the 90 restored sites), large herbivore management, including removal or exclusionary fencing of livestock and/or deer, was deployed and no active planting or seeding occurred. At actively restored sites (51 out of the 90 restored sites), seeding or planting occurred. However, methods were often implemented as combinations of practices including tree and shrub plantings, bioengineering bank stabilization, and reduced or eliminated livestock grazing. The 13 surveyed sites that were not exposed to restoration (out of 103 total sites) were considered reference (control) sites. These sites were chosen based on where local experts indicated that a particular stream reach had vegetation similar in structure and were likely exposed to similar levels of degradation to a project site prior to revegetation activities. These ‘control’ sites were always close to an active restoration site. In control sites, no active exclusion of livestock occurred.

### Data collection

We characterized all sites by the following variables: 1) project design components (four total, described below); 2) site conditions (14 total, described below); and 3) plant cover. Project design components that we measured were: management and revegetation variables such as type of restoration (active, passive, or control), number of original tree species planted, year of restoration implementation, and grazing type (seasonality–including seasonal, multiseasonal, or none; and animal identity–deer only, livestock and deer, or none). Restoration operators and project collaborators provided this information.

To characterize site conditions, we installed three cross-sectional belt transects perpendicular to the channel and stratified within each site at fast-water riffle locations [15]. At these cross-sections we collected data on physical site conditions: landform class, stream width and depth, tree canopy cover (estimated by canopy density and solar radiation), elevation, bank stability and slope, summer flow, and particle size. Stream width and depth was documented using the bankfull width-to-depth ratio and entrenchment [30]. Canopy density (using a densiometer) and solar radiation (using a pathfinder) were measured over the thalweg (deepest point of the channel) at each site. The densiometer data was collected following [31], while the pathfinder data was collected as a percent of solar radiation intercepted by riparian shade. Streambank stability was assessed for both banks at each cross-section according to [32] and bank slope was measured using a clinometer. Summer flow was characterized as either perennial, no flow with standing water in pools, or completely dry. Site characterization also included soil particle size analysis by landform class using standard methods [33]. We collected stream substrate data at each site and calculated percent fine sediment and embeddedness [31]. We used [34] to form our landform class designations, which included the active channel, erosional flood plain, depositional flood plain, and upper bank. We also collected growing season mean, minimum and maximum temperature and precipitation using weather stations that were located at or near each site. In total, we collected data on 14 characteristics at each site.

Plant community data was collected within quadrats (three quadrats per plot), nested within plots (four plots per transect), positioned within belt transects along the three cross-sections [15]. Six 7-m wide belt transects (2 per cross section × 3 cross-sections) continued up each streambank from the thalweg at cross-sections until the upper bank was sampled. Plot location was based on channel morphology at the lowest possible bankfull location (break in slope of a flat depositional surface flooded every 1 to 2 year, on average) and floodprone elevation (2 × bankfull depth) using the 3 independent cross-sections per site [30]. Plot length was variable and based upon the extent of the landform class for each plot (described above). Designations for each plot were based upon field observations of channel morphology and features of aggradation and erosion see [15] for full details.

Relative cover and species richness (common metrics of restoration success [35]) were calculated for seven functional groups (native perennial forbs, native perennial grasses, native ferns, native shrubs, exotic perennial forbs, exotic perennial grasses, exotic annual forbs). These groups were surveyed in quadrats using a modified Daubenmire frame (20 × 50 cm) placed perpendicular to the channel [36]. Species identification followed [37].

### Data analysis

In order to identify differences in success among restored sites, we investigated how restoration design affected revegetation outcomes. Revegetation outcomes were characterized as those related to restoration success including the cover of native functional groups (native ferns, native forbs, and native perennial grasses), total native cover, native species richness, and total invasive species cover e.g. [38]. We used the {nlme} package in R to develop linear mixed effects models predicting these vegetation response variables with the fixed effects of restoration type (control, passive, or active), site age (control sites were marked as 0), planted species number, and grazing type (none, seasonal or multiseasonal), and the random effects of quadrat nested within plot, plot nested within transect, and transect nested within replicate were also included in the model. Different models were developed for each of the seven response variables and all response variables were log transformed to improve normality and homoscedasticity of residuals. Models were initially developed to include all interaction terms; and we then used automated backward stepwise model selection to find the most parsimonious models. Variables were considered for the final models only if their significance level in the full model was <5%. The selection procedure used exact AIC values. Finally, to investigate overall effects of project design on dominant riparian understory functional group cover and richness, we used ANCOVA to understand relationships among revegetation type, site age, and the response variables of (1) cover and richness of native and invasive grasses grouped together and (2) native and invasive forbs grouped together. In cases where significant interactions were found, post-hoc tests were employed, using the {multcomp} package in R.

We then conducted community analyses in order to assess how herbaceous communities differed based on site characteristics and restoration type. We first identified which of the 14 potential explanatory factors might be responsible for community differences. To reduce the pool of potential explanatory variables for the community analysis, we conducted an ordination on our functional group data using a detrended correspondence analysis (DCA). We then subjected the first two DCA axis scores to regression tree analyses (CART, [39]), which used binary recursive partitioning to identify factors potentially driving gradients in plant communities at the functional group level. For the CART analyses, we used the {rpart} and {randomForest} packages in R. We finally performed a PERMANOVA in R to explore how these factors and restoration type affect variation in the functional community data.

## Results

Fifty-three perennial shrubs were found across the sites. Common shrubs found included the invasive Himalayan blackberry (*Rubus discolor*) and the natives California blackberry (*Rubus ursinus*) and Coyote brush (*Baccharis piluaris*). Forty-one perennial trees were identified in our surveys, but the native Arroyo willow (*Salix lasiolepis*) dominated across sites. We identified 131 species of forbs in our surveys (53% native). The dominate forbs at the site included the invasive annuals Poison hemlock (*Conium maculatum*), Italian thistle (*Carduus pycnocephalus*) and Milk thistle (*Silybum marianum*). Finally, we identified 61 species of grass (54% native), but most sites were dominated by the native perennial Creeping wildrye (*Leymus triticoides*).

### Assessing restoration success

**Fig 1:**
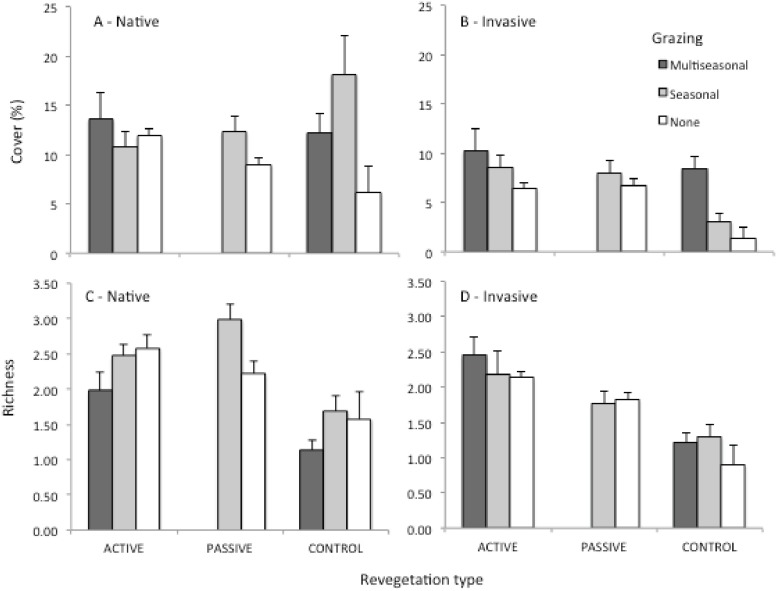
Means ±SE of cover of herbaceous native plant species (A) and invasive plant species (B), and richness of native plant species (C) and invasive plant species (D) across restoration and grazing types.

Final linear mixed effects models for all response variables can be found in Table 1. Passive (mean = 12.32, SD = 14.85) and active (mean = 10.8, SD = 17.0) restoration resulted in significantly lower cover of native species compared to control (mean = 18.16, SD = 25.48) in the presence of seasonal grazing (Estimate = -0.61, SE = 0.32, t = -1.92, p = 0.05, Fig 1A). Native richness was also lower in control plots (mean = 1.36, SD = 1.4), compared to active restoration (mean = 2.52, SD = 3.5; Estimate = -0.53, SE = 0.23, t = -2.28, p = 0.02, Fig 1C). Invasive species cover was not significantly different across treatments (Fig 1B), however, invasive species richness was lower overall in the control plots (mean = 1.19, SD = 1.15) compared to active restoration plots (mean = 2.16, SD = 2.19; Estimate = -0.58, SE = 0.19, t = -3.04, p = 0.003, Fig 1D).

**Table 1:** Final models of all response variables with AIC values and AIC values of full model (all factors and all interactions included) for comparison. Explanatory variables include: Type (active, passive or no restoration); Graze (absence or presence of grazing); Age (number of years after the restoration project has been implemented); Species (number of species planted for restoration). All models included a random factor of quadrat nested within plot, plot nested within transect, and transect nested within replicate.

Response variable	Model	Model AIC	Full model AIC
Native fern cover	Graze + Age + Species	4110	4159
Native forb cover	Graze*Species	4777	4822
Native perennial grass cover	Type + Graze	5118	5172
Total native cover	Type*Graze	5241	5298
Native species richness	Type	4234	4290
Invasive species cover	Revegetation type + Graze + Species	5127	5164
Invasive species richness	Type	4247	4310

We found that older restoration sites had less native fern cover (Estimate = -1.47, SE = 0.40, t = -3.69, p < 0.001) and that native fern cover declined with increasing numbers of species planted (Estimate = -2.25, SE = 0.84, t = -2.68, p = 0.009, data not shown). In the presence of seasonal grazing, we found very low cover of native ferns across both site age and the number of planted tree species. In the absence of grazing, these negative relationships had a steeper slope (Estimate = -1.46, SE = 0.40, t = -3.69, p < 0.001). We also found that the cover of native forbs were unaffected by all factors except for the interaction between planted species number and grazing. In the absence of grazing, we found no significant relationship between cover of native forbs and number of planted tree species, but in the presence of grazing; there was a negative relationship (Estimate = -2.28, SE = 1.13, t = -2.01, p = 0.04). Finally, none of the factors contributed to differences in native perennial grass cover.

**Fig 2:**
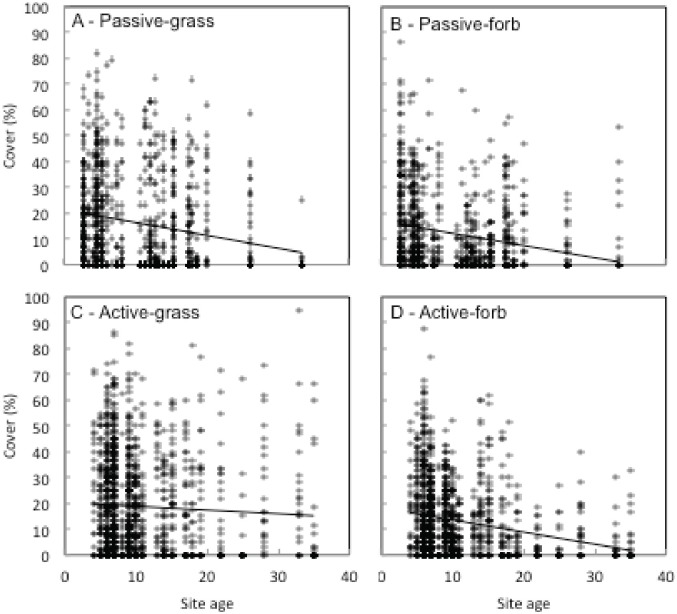
Relationships between site age and percent cover of (A) grasses in passive restoration sites, (B) forbs in passive sites, (C) grasses in active sites, and (D) forbs in active restoration sites.

**Fig 3:**
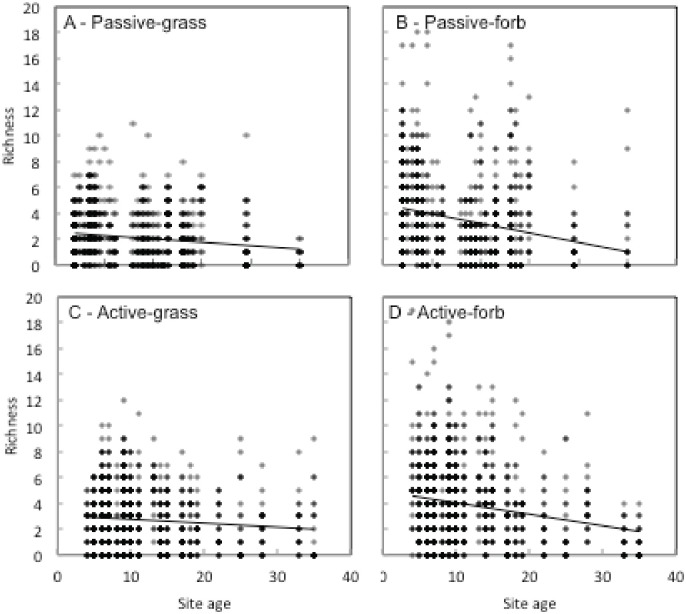
Relationships between site age and species richness of (A) grasses in passive restoration sites, (B) forbs in passive sites, (C) grasses in active sites, and (D) forbs in active restoration sites.

The ANCOVA showed a significant interaction was found between revegetation type and site age for both grass cover (F = 4.14, p = 0.04) and forb cover (F = 10.03, p = 0.002; Fig 2). Grass and forb cover demonstrated a negative relationship with site age in passively restored sites (p = 0.05 for both groups), but only forb cover demonstrated a negative relationship with site age in actively restored plots (p = 0.05). A significant interaction was also found between revegetation type and site age for grass richness (F = 4.54, p = 0.03) only. Specifically, we detected a negative association between grass richness and site age in passively restored sites (p < 0.001). We also detected a negative association between forb richness and site age overall (F = 172.97, p <0.001; Fig 3).

### Describing plant communities

The DCA indicated a strong landform gradient in the plant community (S1 Fig) where the upper banks were dominated by annual grasses and forbs, while functional groups of exotic perennial grasses and forbs dominated the channels. The regression tree predicting DCA axes converged on a final set of potential explanatory variables to include in further analysis, which included (in descending order of importance): percent soil fines, percent shade, landform type, and summer flow. These variables explained 48% of the variation in DCA axes. As might be expected from this analysis, PERMANOVA results showed the factors of landform type (F = 3.14, p = 0.001), percent shade (F = 5.80, p = 0.001), summer flow (F = 5.38, p = 0.001) and the interaction between restoration type and percent fines (F = 5.77, p = 0.001) were important for driving differences in the understory functional community.

## Discussion

Considering how profoundly vegetation communities can change following management of riparian systems [29, 38], we expected that herbaceous riparian plant communities would respond to restoration type [28]. However, riparian zones are often composed of species that are simultaneously tolerant of dynamic and heterogenous resource availability [40–41, 27], which can make them less responsive to biotic drivers [42]. We found that, similar to other studies [27, 40], the abiotic factors of landform type, percent shade, and summer flow were the primary determinants of understory plant communities, irrespective of restoration type. The absence of an interaction between abiotic factors and restoration type could be driven by scale dynamics. Spatial scale has implications for restoration outcomes because factors that drive survival dynamics of planted or protected plant species differ across regional, local, and microsite scales [43–44]. Abiotic factors that typically regulate riparian plant community structure operate at larger spatial scales than restoration approaches such as tree plantings and grazing exclosures [45].

Similar to what we identified in this work, other studies have found an absence of an apparent relationship between factors associated with restoration activities (e.g. site age, planted species number and presence of subsequent grazing) and native riparian plant species [25, 46]. From a mechanistic perspective, it is possible that clear signals of restoration effects on native grass vegetation were masked by the dynamic nature of the system. Riparian habitats are non-equilibrium systems where frequent disturbance from fluvial and hydrological processes create an extremely dynamic herbaceous plant community [27, 42]. These disturbance events could effectively ‘reset’ the system seasonally by swamping any differences in the herbaceous plant community arising from post-restoration management effects e.g. [47].

In some cases, we did find lower native plant cover and species richness in passive compared to active restorations. Passive restoration is expected to enhance native biodiversity by providing woody and herbaceous seedlings opportunities to establish in the absence of grazing pressure [48]. However, low intensity grazing can maintain and even enhance native herbaceous cover and diversity by creating niche space through grazing activities that reduce competition, and provide resources through microsite disturbance [49–51]. Some studies have documented a decline in native plant species richness following grazing exclusion that was likely a response to weed invasion [52–54] or reorganization of dominant species identity [29, 48]. Other studies have found that grazing cessation in riparian systems lead to a dominance of woody species, which can negatively affect native understory plants e.g. [55–56]. This collective body of work suggests that plant communities should be carefully monitored after the deployment of passive restoration efforts.

Ferns are an important component of riparian systems, providing shade, habitat, and sometimes nitrogen to bank and stream areas and they are typically found in undisturbed riparian areas e.g. [57]. We found a negative relationship between the number of tree species planted for restoration and native fern cover, which could suggest that tree species abundance correlates in some way to disturbance intensity of the revegetation effort. We also found a strong negative relationship between native fern cover and restoration age. Ferns tend to prefer species-poor, low productivity riparian sites [58]. Species richness and productivity is expected to scale with restoration site age, resulting from the establishment and growth of planted or protected seedlings at a revegetation site. This woody growth decreases light availability and increases the buildup of sediment; two factors that limit fern growth [59]. In cases where fern cover enhancement is desired, thinning overstory foliage or planting fewer trees closest to the riparian bank edge could be an effective technique.

Grazing pressure can also modify herbaceous plant community composition in riparian systems [50]. For example, after grazing is excluded from a riparian site, the herbaceous plant community can become dominated by less disturbance tolerant, more hydrophytic species [29]. Alternatively, livestock can maintain species composition while modifying relative abundance [51] through preference grazing. Either of these scenarios might lead to our findings of a negative relationship between native forb cover and the number of tree species planted for restoration in the presence of grazing [60].

### Implications for management

Where soil stabilization is desired in the absence of planting, weed management strategies should be employed in tandem with passive restoration to limit weed invasion and subsequently enhance understory native plant communities. Employing passive restoration in an adaptive management framework would also be an effective way to increase the likelihood that understory plant communities are developing along desirable trajectories. Alternatively, when active restoration is used in an area grazed by livestock, forb cover should be monitored for several years after planting to ensure adequate cover for soil protection. Finally, our results suggest that when biotic factors (such as cover of invasive species) are used for riparian restoration assessment, focusing on vegetation less affected by abiotic dynamics, such as trees and woody shrubs, might be the most effective for illustrating restoration outcomes.

Although some of our results suggest that active restoration will provide more utility for native species enhancement in degraded riparian areas compared to passive approaches, this work also highlights some of the context-dependency that can mediate restoration outcomes e.g. [10]. Since the effects of passive restoration can be quite rapid [14], this approach might be more useful than active restoration in situations where fast dominance of pioneer species is required to arrest major soil loss through erosion. As a result, we caution against labeling one restoration technique as superior to another. Managers should identify ideal restoration outcomes in the context of historic and current site characteristics (as well as a range of acceptable alternative states) and choose restoration approaches that best facilitate the achievement of revegetation goals.

## Supporting information

S1 Fig Detrended correspondence analysis. (EPS)
